# Efficacy and safety of tranexamic acid in reducing hidden blood loss during unilateral total knee arthroplasty: a retrospective study

**DOI:** 10.3389/fmed.2025.1552893

**Published:** 2025-06-03

**Authors:** Luo Liangliang, Huang Wei, Zhang Tao, Pan Pin, Hu Lianying

**Affiliations:** Department of Orthopedics, The Second People’s Hospital of Hefei, Hefei Hospital Affiliated to Anhui Medical University, Hefei, Anhui, China

**Keywords:** tranexamic acid (TXA), total knee arthroplasty (TKA), hidden blood loss, osteoarthritis, efficacy and safety

## Abstract

This retrospective study evaluates the efficacy and safety of tranexamic acid (TXA) in reducing hidden blood loss during unilateral total knee arthroplasty (TKA) in patients with osteoarthritis. As the aging population leads to a rise in degenerative knee joint diseases, TKA has become a common surgical intervention. However, it is often associated with significant hidden blood loss, accounting for 60–75% of total blood loss, which can result in complications such as anemia and increased transfusion needs. Our study included 123 patients who underwent TKA between June 2019 and June 2022, divided into an observation group receiving TXA and a control group receiving saline. TXA was administered intravenously before surgery and locally into the joint cavity post-incision. The study found that TXA significantly reduced total blood loss by 37.4%, with a notable decrease in hidden blood loss (*p* < 0.05). The TXA group also exhibited lower transfusion rates (27.4 vs. 45.9%, *p* = 0.033) and reduced intraoperative blood loss (330.6 ± 25.3 mL vs. 494.4 ± 32.8 mL, *p* < 0.001). Importantly, TXA did not increase the risk of thromboembolic complications, with no significant differences in deep vein thrombosis or pulmonary embolism between the groups. Coagulation parameters remained stable, supporting TXA’s safety profile. These findings suggest that TXA is a safe and effective strategy for managing blood loss in TKA, potentially improving patient outcomes, reducing healthcare costs, and optimizing resource utilization.

## 1 Introduction

As the population ages, the incidence of degenerative knee joint disease is rising, significantly impacting patients’ daily activities and increasing the socioeconomic burden (1). Total knee arthroplasty (TKA) is now widely used to treat this condition, as it effectively relieves pain, restores function, and improves patients’ quality of life (2, 3). However, TKA is often associated with considerable blood loss (1000–1,500 mL) (4, 5). Although the visible blood loss during surgery is minimal, the hidden blood loss—unmeasured perioperative blood loss often caused by tissue infiltration or undetected bleeding—is significant, comprising approximately 60–75% of the total blood loss (6–8). Hidden blood loss can lead to anemia, hypoxemia, delirium, pulmonary embolism, and other complications, which can have severe consequences, especially in the elderly population (7–10). To address anemia, blood transfusions are often necessary, but large-volume transfusions increase surgical risks and can lead to transfusion-related complications, such as viral infections, hemolytic reactions, and immune responses (11, 12). To reduce the volume and rate of transfusions, methods such as autologous blood transfusion, erythropoietin administration, intraoperative hemodilution, autologous blood salvage, and the use of anticoagulants are employed (13). Among these, the intraoperative use of TXA has been proven to reduce blood loss and lower transfusion rates in orthopedic surgeries (7, 8, 14, 15). TXA is a synthetic derivative of the amino acid lysine and functions as an antifibrinolytic drug. It works by competitively inhibiting the lysine-binding sites on plasminogen molecules, effectively preventing fibrinolysis (16). As a reversible blocker of these binding sites, TXA plays a crucial role in stabilizing blood clots. TXA has demonstrated effectiveness in various surgical settings, and its use has been associated with decreased bleeding and lower rates of blood transfusions, making it a valuable tool in managing surgical and traumatic hemorrhage (17). However, there is currently no research on whether TXA reduces hidden blood loss during the perioperative period of TKA.

This article aims to study the efficacy and safety of intravenous TXA treatment in patients with osteoarthritis undergoing unilateral TKA, and it will also conduct a quantitative analysis of TXA’s effect on reducing hidden blood loss.

## 2 Materials and methods

### 2.1 General information

**Figure 1:**
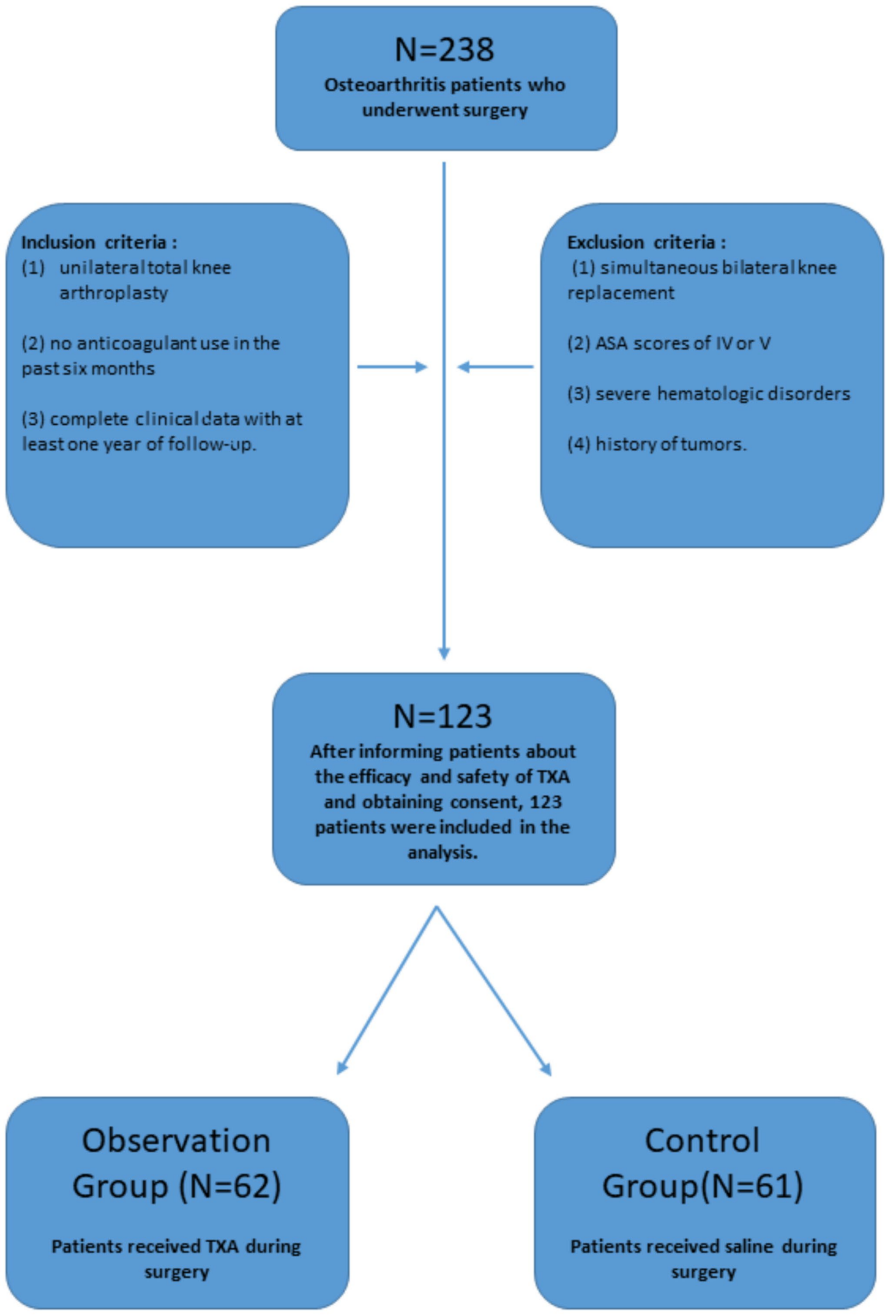
Patient selection flowchart. A total of 238 patients with osteoarthritis who underwent surgical treatment at our hospital between June 2019 and June 2022 were initially considered for the study. Following the exclusion process, 123 patients met the eligibility criteria. These patients were then divided into two groups based on the use of TXA: the observation group (*n* = 62) and the control group (*n* = 61). ASA, American Society of Anesthesiologists; TXA, tranexamic acid.

Our study was reviewed and approved by the Medical Ethical Committee of our hospital. We examined 238 patients with osteoarthritis who underwent surgical treatment at our hospital from June 2019 to June 2022. The inclusion criteria were: (1) unilateral TKA, (2) no use of anticoagulant medication in the past 6 months, and (3) availability of complete clinical data with the ability to undergo at least 1 year of follow-up. The exclusion criteria were: (1) simultaneous bilateral knee replacement, (2) American Society of Anesthesiologists (ASA) scores of IV or V, (3) severe hematologic disorders, and (4) history of tumors. After informing patients and their families about the efficacy and safety of TXA and obtaining their consent, a total of 123 patients were included in the analysis. Among these, 62 patients received TXA treatment during surgery (observation group), while 61 patients received saline as a control (control group) (Figure 1).

### 2.2 Methods

#### 2.2.1 Treatment methods

Both groups underwent unilateral TKA using an identical surgical procedure, with all surgeries performed by the same surgeon. General anesthesia was administered to all patients. After successful induction of anesthesia, patients were positioned supine, disinfected, and draped under sterile conditions. A tourniquet was inflated to 60 kPa. A midline longitudinal incision was made, extending approximately 6 cm above the patella and continuing to about 20 cm along the medial edge of the tibial tuberosity. The skin, subcutaneous tissue, and fascia were incised sequentially. The patella was everted laterally, part of the fat pad was excised, and the joint capsule was sharply dissected to fully expose the knee joint. The joint cavity was thoroughly cleaned, and soft tissues were released. The femoral and tibial bone ends were prepared, and trial implants were fitted. Bone surfaces were processed, and bone cement was mixed to the appropriate consistency. The prostheses were implanted with slight pressure and maintained until the cement solidified, ensuring full contact between the prosthesis, bone cement, and bone surface. Excess bone cement was removed, and knee joint stability was assessed. The incision was irrigated repeatedly with 5,000 mL of saline and sterile irrigation solution. The tourniquet was then released, and hemostasis was carefully achieved. After confirming the correct count of instruments and gauzes, a drainage tube was placed, and the incision was closed in layers and dressed with sterile materials.

Patients in the observation group received an intravenous infusion of TXA injection (1.0 g/100 mL, Hunan Dongting Pharmaceutical Co., Ltd., China) starting 30 min before surgery. After suturing the incision, 1.0 g of tranexamic acid injection was administered locally into the joint cavity (18). Patients in the control group received an equal volume of normal saline at the same time points.

Both groups received the same postoperative treatment measures, including blood transfusion, volume expansion, and maintenance of electrolyte balance. Patients began oral rivaroxaban tablets 10 mg once daily (Xarelto, Bayer Germany) for anticoagulation on the second day after surgery, continuing for 2 weeks. Routine Doppler ultrasound was used to check for deep vein thrombosis (DVT) in the lower extremities. If pulmonary embolism (PE) symptoms were suspected, enhanced CT scans of the lungs were performed for diagnosis. Blood transfusions were administered when hemoglobin levels fell below 90 g/L or if anemia symptoms (such as poor mental state, palpitations, or shortness of breath) were present.

#### 2.2.2 Observation indicators

##### 2.2.2.1 General conditions and surgery-related factors

For all patients, we recorded general information including age, gender, height, and weight, as well as surgery-related factors such as operation time, intraoperative blood loss, transfusion volume, postoperative hospital stay, and follow-up duration. Hemoglobin (Hb), hematocrit (Hct), and coagulation indicators (D-dimer, PT, APTT, INR) were measured for each patient on the morning of the surgery and again 72 h postoperatively (8). Visible blood loss (VBL) refers to the total amount of blood loss during the perioperative period, which includes intraoperative blood loss and postoperative drainage volume. Intraoperative blood loss was calculated from the blood collected in suction bottles and weighed swabs. The drainage tube was removed 48 h after surgery, and the total drainage volume was recorded as part of the visible blood loss.

##### 2.2.2.2 Blood loss calculation

**Figure 2:**
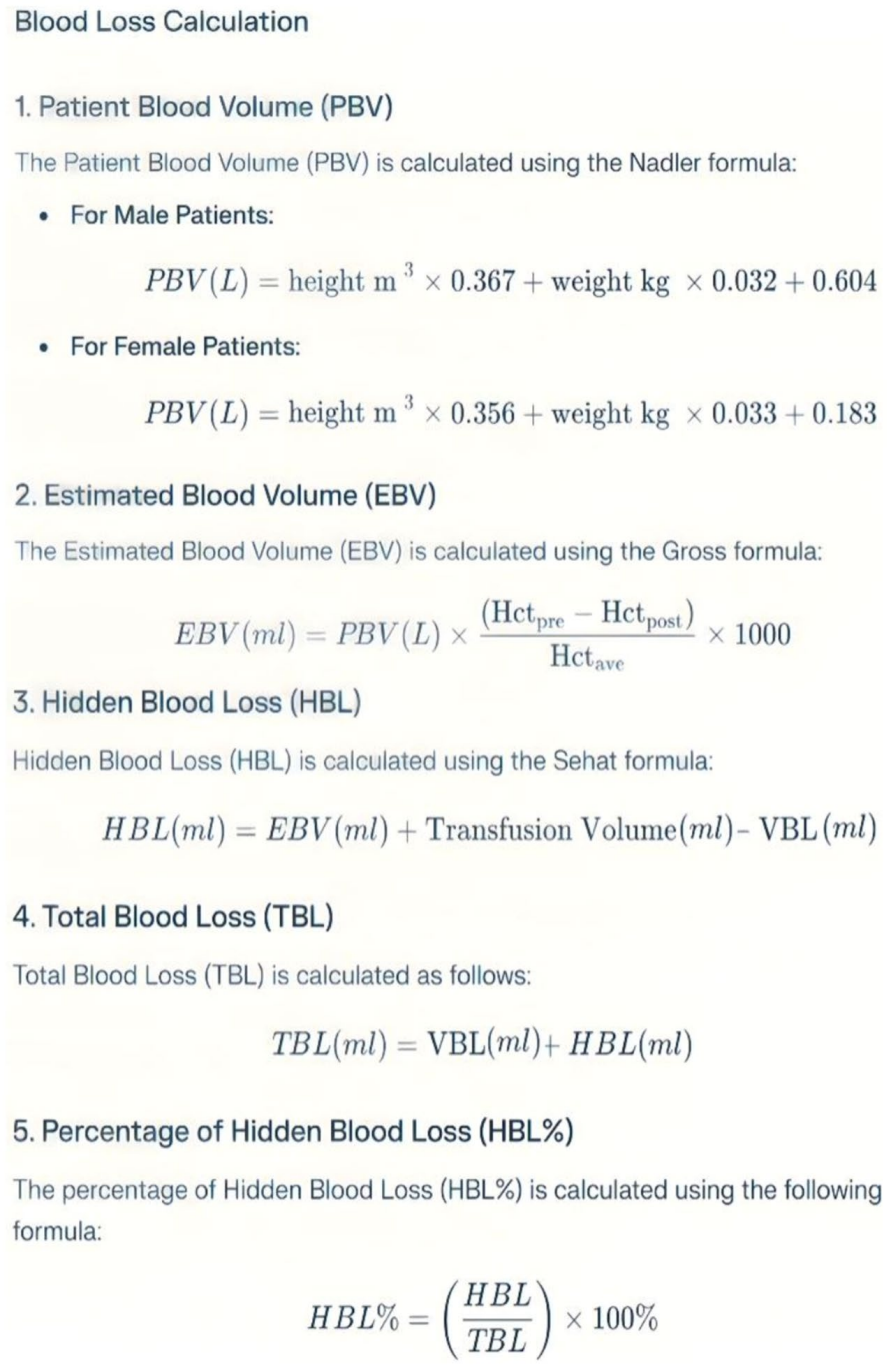
Blood loss calculation. Hctpre, Preoperative hematocrit; Hctpost, Hematocrit at 72 h postoperatively; Hctave, Average of Hctpre and Hctpost; VBL, Visible blood loss = Intraoperative blood loss + Drainage volume.

The Patient Blood Volume (PBV) was calculated using the Nadler formula (19), while the Estimated Blood Volume (EBV) was determined using the Gross formula (20). Hidden Blood Loss (HBL) was assessed with the Sehat et al. (21) formula. Furthermore, Total Blood Loss (TBL) was calculated, along with the percentage of Hidden Blood Loss (HBL %) (Figure 2).

### 2.3 Statistical methods

All statistical comparisons were conducted using SPSS version 26.0 (SPSS Inc., USA). Continuous variables were analyzed using the Wilcoxon Mann–Whitney U test or the independent sample *t*-test. For categorical variables, Pearson’s chi-square test or Fisher’s exact test was employed. A *p* < 0.05 was considered statistically significant.

## 3 Results

### 3.1 Comparison of general characteristics

The preoperative general characteristics of patients in both groups, including age, gender, height, weight, and ASA score, were compared, revealing no statistically significant differences (*p* > 0.05). This indicates that the two groups were comparable (Table 1).

**Table 1:** Characteristics of the patients.

	Observation group	Control group	*p*-value
Age (years)	69.1 ± 9.9	68.7 ± 9.2	0.81
Gender			
Female, *n* (%)	48 (77.4)	45 (73.8)	0.64
Male, *n* (%)	14 (22.6)	16 (26.2)	
Height (cm)	166.5 ± 9.5	168.9 ± 9.9	0.18
Weight (kg)	56.0 ± 12.1	57.8 ± 10.7	0.69
BMI	21.5 ± 3.0	20.4 ± 3.8	0.47
ASA grade			0.58
Grade I, *n* (%)	16 (25.8)	14 (23.0)	
Grade II, *n* (%)	18 (29.0)	17 (27.8)	
Grade III, *n* (%)	28 (45.2)	30 (49.2)	

No significant difference among the characteristics of the patients in the two groups was observed (*P* > 0.05).

### 3.2 Comparison of perioperative data

All surgeries were performed by the same surgeon. There were no statistically significant differences between the two groups regarding operation time, postoperative hospital stay, and follow-up duration (*p* > 0.05). However, intraoperative blood loss in the observation group (330.6 ± 25.3 mL) was significantly lower than that in the control group (494.4 ± 32.8 mL) (*p* < 0.001) (Table 2).

**Table 2:** Perioperative parameters of the patients.

	Observation group (*n* = 62)	Control group (*n* = 61)	*P-*value
Operation time (min)	93.1 ± 10.0	90.7 ± 8.7	0.18
Intraoperative blood loss (ml)	330.6 ± 25.3	494.4 ± 32.8	<0.001
Post-operative hospitalization time (day)	7.8 ± 0.7	7.7 ± 1.1	0.81
Follow up period (month)	23.1 ± 7.1	21.1 ± 6.4	0.10

### 3.3 Comparison of blood loss and transfusion

The observation group exhibited lower intraoperative blood loss, postoperative drainage volume, visible blood loss (VBL), hidden blood loss (HBL), and total blood loss (TBL) compared to the control group (*p* < 0.05). The transfusion volume in the observation group (261.5 ± 135.6 mL) was significantly less than that in the control group (345.3 ± 152.1 mL) (*p* = 0.023). Additionally, the transfusion rate in the observation group was 27.4%, which was lower than the control group’s rate of 45.9% (*p* = 0.033) (Table 3).

**Table 3:** Perioperative parameters regarding hidden blood loss.

	Observation group (*n* = 62)	Control group (*n* = 61)	*P-*value
Intraoperative blood loss (ml)	330.6 ± 25.3	494.4 ± 32.8	<0.001
Total volume of drains (ml)	192.8 ± 41.9	260.9 ± 50.5	<0.001
Preoperative Hb values (g/L)	122.6 ± 12.7	120.6 ± 13.5	0.40
Postoperative Hb values (g/L)	100.5 ± 14.0	93.0 ± 13.5	0.003
Preoperative Hct values (%)	36.6 ± 3.5	35.8 ± 4.0	0.80
Postoperative Hct values (%)	30.5 ± 4.1	26.8 ± 3.9	<0.001
PBV(ml)	3993.0 ± 590	4,094 ± 675	0.38
EBV(ml)	730.75 ± 151.8	1199.3 ± 171.3	<0.001
VBL(ml)	522.3 ± 128.8	754 ± 144.9	<0.001
HBL(ml)	645.7 ± 228.9	1257.0 ± 170.7	<0.001
TBL(ml)	1143.9 ± 717.1	1825.1 ± 747.8	<0.001
HBL %	58.7 ± 17.7	67.7 ± 22.9	0.003
Volume of transfusion (mL)	261.5 ± 135.6	345.3 ± 152.1	0.023
Transfusion (*n*, 100%)	17 (27.4%)	28 (45.9%)	0.033

Hb, Hemoglobin; Hct, Hematocrit; PBV, patient blood volume; EBV, estimated blood loss volume; VBL, visible blood loss; HBL, hidden blood loss; TBL, total blood loss; HBL%, the percentage of HBL.

### 3.4 Comparison of coagulation indicators

In the observation group, there were no statistically significant differences in platelet count, PT, APTT, INR and values before and after surgery. Comparisons between the two groups also showed no statistically significant differences (*p* > 0.05). Both groups exhibited an increase in D-dimer levels postoperatively (*p* < 0.05), with the control group showing a more significant increase compared to the observation group (*p* < 0.05) (Table 4).

**Table 4:** Comparison of coagulation indicators between the two groups.

	Observation group		Control group	
	Before	After	Before	After
Platelet count (10^9^)	119.6 ± 77.3	122 ± 67.8	121.8 ± 66.7	120.5 ± 68.9
D-dimer (mg/L)	0.32 ± 0.21	2.4 ± 1.9^#*^	0.28 ± 0.17	4.5 ± 2.9^#^
PT (s)	12.7 ± 0.8	13.8 ± 0.6	12.1 ± 0.9	14.1 ± 0.6
APTT (s)	34.2 ± 3.8	36.1 ± 4.3	34.1 ± 4.1	35.8 ± 3.2
INR (s)	1.2 ± 0.12	1.3 ± 0.11	1.1 ± 0.22	1.3 ± 0.14

^#^Compared with before treatment, *P* < 0.05. *Compared with Control Group, *P* < 0.05.

### 3.5 Comparison of postoperative complications

Complications during the follow-up period were recorded for both groups. No statistically significant differences were observed between the two groups regarding deep vein thrombosis (DVT), pulmonary embolism (PE), wound complications, infections, periprosthetic fractures, and prosthesis displacement (*p* > 0.05) (Table 5).

**Table 5:** Postoperative complications of two groups.

	Observation group	Control group	*P*-value
Deep venous thrombosis (*n*, 100%)	3 (4.8)	4 (6.6)	0.69
Pulmonary embolism (*n*, 100%)	0 (0)	0 (0)	
Wound complications (*n*, 100%)	2 (3.2)	2 (3.3)	0.62
Infection (*n*, 100%)	2 (3.2)	3 (4.9)	0.68
Periprosthetic fractures (*n*, 100%)	0 (0)	0 (0)	
Dislocation (*n*, 100%)	0 (0)	0 (0)	

## 4 Discussion

The rising prevalence of knee osteoarthritis has led to a significant global increase in TKA procedures (1–3, 22). While TKA is effective in treating end-stage knee osteoarthritis, providing pain relief and improved functionality, it is also associated with substantial perioperative blood loss (4–6). This blood loss can lead to various complications, including anemia, increased need for transfusions, and prolonged hospital stays, all of which may negatively impact patient outcomes and drive up healthcare costs. As a result, effective blood management strategies during TKA have become a crucial focus in orthopedic research and clinical practice. Our study demonstrates that the use of TXA both intraoperatively and preoperatively can significantly reduce total blood loss (TBL) by 37.4% compared to the control group. This finding aligns with numerous meta-analyses and randomized controlled trials that consistently support the efficacy of TXA in reducing perioperative blood loss in TKA (5, 10, 23, 24). TXA is an antifibrinolytic drug widely used in various types of surgeries, including cardiac, liver, spine, and hip and knee replacement surgeries. It has demonstrated good efficacy without increasing the incidence of deep vein thrombosis (DVT) or other related complications. TXA works by reducing the physiological process of fibrinolysis and preventing fibrin degradation while inhibiting platelet-activating factors, thereby protecting platelets. In TKA, surgical trauma stimulates the fibrinolytic process, and the use of a tourniquet can expose the limb to anaerobic conditions, causing vascular endothelial tissue to release plasminogen activators, which promote fibrinolysis and increase postoperative blood loss (23–25). The application of TXA can counteract this fibrinolytic response during TKA, thereby reducing blood loss.

### 4.1 Reduction in hidden blood loss

Our study’s most significant finding is the substantial reduction in hidden blood loss (HBL) achieved through the administration of TXA. In the control group, HBL constituted 67.7% of the total blood loss (TBL), underscoring the need to address this often-overlooked component in TKA. The concept of hidden blood loss, first introduced by Sehat et al. (6), has gained increasing attention in orthopedic surgery because it represents a significant portion of TBL that is not immediately apparent during or after surgery. Our findings indicate that TXA treatment significantly reduced both the volume and percentage of HBL (*p* < 0.05). This reduction is crucial, as hidden blood loss can lead to postoperative anemia, delayed rehabilitation, and increased risk of complications (7–10, 26). TXA likely reduces HBL by stabilizing clots within tissue spaces and decreasing ongoing fibrinolysis in the immediate postoperative period.

### 4.2 Reduction in visible blood loss and transfusion requirements

Beyond its impact on hidden blood loss, TXA effectively reduced intraoperative blood loss and postoperative drainage, which are components of visible blood loss. This comprehensive reduction resulted in significantly lower transfusion rates in the TXA group compared to the control group (27.4 vs. 45.9%, *p* < 0.05). This outcome aligns with other studies which reported similar reductions in transfusion needs with TXA use (26, 27). Reducing transfusion rates is particularly important due to the potential risks associated with allogeneic blood transfusions, including allergic reactions, transfusion-related acute lung injury, and the transmission of infectious diseases. Additionally, reducing transfusion requirements can lead to significant cost savings for healthcare systems and improved resource allocation (14, 15, 28, 29).

### 4.3 Safety profile of tranexamic acid

A primary concern with using antifibrinolytic agents like TXA in orthopedic surgery is the potential increase in thromboembolic complications, such as deep vein thrombosis (DVT) and pulmonary embolism (PE). Our study found no significant difference in DVT incidence between the TXA and control groups (4.8 vs. 6.6%), and notably, no cases of PE occurred in either group. These findings support the growing body of evidence, including recent meta-analyses, indicating that intravenous TXA administration in TKA does not increase the risk of thromboembolic events (5, 14, 17, 28).

The safety of TXA in TKA can be attributed to its mechanism of action, which primarily affects the fibrinolytic system rather than the coagulation cascade. TXA inhibits the activation of plasminogen to plasmin, thereby preventing the breakdown of fibrin clots. This mechanism allows for improved hemostasis without significantly altering the balance of the coagulation system.

### 4.4 Effects on coagulation parameters

Our study observed that both groups showed increased D-dimer levels postoperatively, which is expected following major orthopedic surgery. However, the increase was less pronounced in the TXA group, suggesting TXA’s inhibitory effect on postoperative fibrinolysis. This finding is important as it demonstrates that TXA effectively modulates the fibrinolytic response without completely suppressing it, potentially maintaining a balance between hemostasis and normal fibrinolytic activity. Importantly, other coagulation parameters, including platelet count, prothrombin time (PT), activated partial thromboplastin time (APTT), and international normalized ratio (INR), showed no significant changes or differences between the groups. This further supports the safety profile of TXA, indicating that its use does not adversely affect the overall coagulation status of patients undergoing TKA.

### 4.5 Clinical implications

The findings of our study have several important clinical implications. First, the significant reduction in total blood loss, hidden blood loss, and transfusion requirements achieved with TXA use can lead to improved patient outcomes, including faster recovery, reduced risk of anemia-related complications, and potentially shorter hospital stays. Second, the demonstrated safety profile of TXA in our study supports its routine use in TKA for patients without specific contraindications. Moreover, the reduction in transfusion rates associated with TXA use has potential economic benefits for healthcare systems. Allogeneic blood transfusions are costly, and reducing their frequency can lead to significant cost savings. Additionally, by reducing the need for transfusions, TXA use may help alleviate pressure on blood bank resources, which is particularly relevant in settings where blood products may be in limited supply.

### 4.6 Limitations and future directions

While our study offers valuable insights into the efficacy and safety of TXA in TKA, several limitations warrant attention in future research. Firstly, although we did not observe an increase in thromboembolic complications with TXA use, longer-term follow-up studies are necessary to evaluate any potential late-onset complications. Secondly, future studies should examine the efficacy and safety of TXA in high-risk patient populations, such as those with a history of thromboembolic events or other comorbidities that might influence bleeding or clotting risks. Thirdly, as a retrospective study, it is inherently prone to biases such as recall, selection, observer, and information bias, which may compromise the validity and generalizability of the findings. Lastly, investigating the potential synergistic effects of TXA in combination with other blood conservation strategies, such as autologous blood salvage techniques or the use of erythropoiesis-stimulating agents, could offer a more comprehensive approach to blood management in TKA.

## 5 Conclusion

Our study demonstrates that administering TXA twice—first intravenously before surgery and then topically during TKA—effectively reduces unilateral total perioperative blood loss, hidden blood loss, and transfusion requirements without increasing the risk of thromboembolic complications. These findings endorse TXA as a safe and effective strategy for blood management in TKA.

## Data Availability

The datasets presented in this study can be found in online repositories. The names of the repository/repositories and accession number(s) can be found in the article/Supplementary material.
